# The genome sequence of the common frog, *Rana temporaria* Linnaeus 1758

**DOI:** 10.12688/wellcomeopenres.17296.1

**Published:** 2021-10-22

**Authors:** Jeffrey W. Streicher, Wellcome Sanger Institute Tree of Life programme, Wellcome Sanger Institute Scientific Operations: DNA Pipelines collective, Tree of Life Core Informatics collective, Darwin Tree of Life Consortium

**Affiliations:** 1 Department of Life Sciences, Natural History Museum, London, UK

**Keywords:** Rana temporaria, common frog, genome sequence, chromosomal

## Abstract

We present a genome assembly from an individual female *Rana temporaria *(the common frog; Chordata; Amphibia; Anura; Ranidae). The genome sequence is 4.11 gigabases in span. The majority of the assembly is scaffolded into 13 chromosomal pseudomolecules. Gene annotation of this assembly by the NCBI Eukaryotic Genome Annotation Pipeline has identified 23,707 protein coding genes.

## Species taxonomy

Eukaryota; Metazoa; Chordata; Craniata; Vertebrata; Euteleostomi; Amphibia; Batrachia; Anura; Neobatrachia; Ranoidea; Ranidae; Rana; *Rana temporaria* Linnaeus 1758 (NCBI:txid8407).

## Introduction

The common frog, *Rana temporaria* (Anura: Ranidae), is widely distributed throughout Europe. It has a biphasic life cycle that includes aquatic, benthic larvae and terrestrial (sometimes semi-aquatic) adults. In the United Kingdom, populations of *R. temporaria* breed as early as late January with most tadpoles metamorphosing in June or July, however, tadpoles occasionally overwinter ( Walsh *et al.*, 2016). The common frog is an emerging model for the study of genetic sex determination, as different populations vary in their degree of sex chromosome differentiation (e.g. ( Phillips *et al.*, 2020)). 

The nuclear genome size of *R. temporaria* was previously estimated to be between 3.31 and 4.91 picograms (= 3.24 and 4.80 gigabases; ( Gregory, 2021)) which is consistent with our 4.11 gigabase assembly. The thirteen pseudomolecules in our assembly match the expected number of chromosomes in *R. temporaria* (2N = 26; five macro- and eight micro-chromosomes; ( Spasić-Bošković *et al.*, 1997). This is the second nuclear genome sequence to be reported from a ranid anuran ( Hammond *et al.*, 2017).

The *R. temporaria* reference genome sequence from a UK-collected individual will provide a useful resource for enhancing and further interpreting available datasets including transcriptomic data that document the immune response of *R. temporaria* to the amphibian diseases caused by *Batrachochytrium dendrobatidis* and *Ranavirus* ( Price *et al.*, 2015).

## Genome sequence report

**Figure 1.**
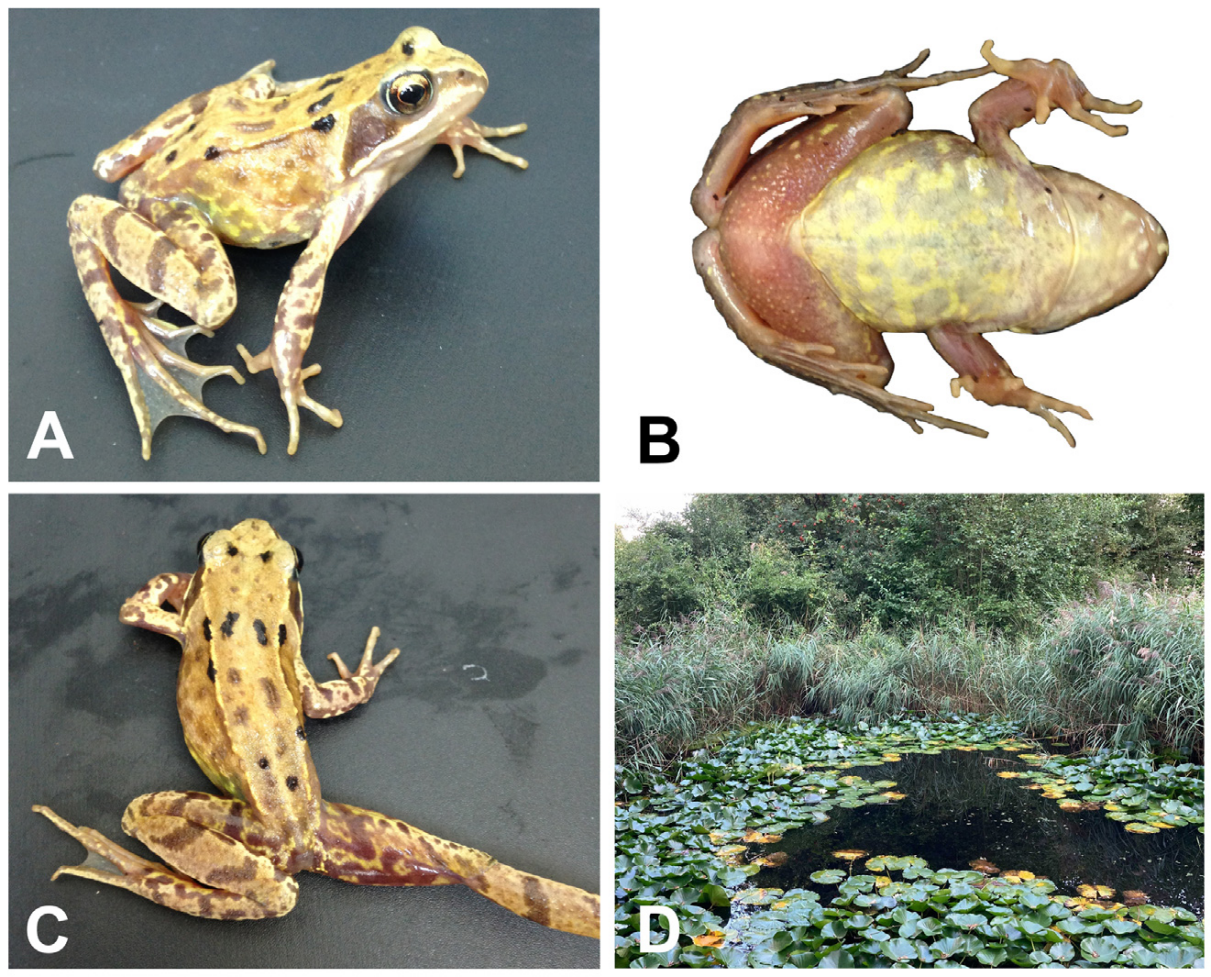
Images of the *Rana temporaria* specimen sequenced. ( **A**) Female voucher specimen of *R. temporaria* (BMNH 2013.483; Field ID, JWS 757; Snout–Vent Length 49.2 mm) from which the genome was sequenced. ( **B**) Ventral surface of NHMUK 2013.483. ( **C**) Dorsal and posterior thigh surfaces of NHMUK 2013.483. ( **D**) The individual was collected from the Natural History Museum Wildlife Garden, London, England.

The genome was sequenced from one female *R. temporaria* ( Figure 1A–C) collected from The Natural History Museum Wildlife Garden, London, UK ( Figure 1D. A total of 63-fold coverage in Pacific Biosciences single-molecule long reads (N50 27 kb) and 51-fold coverage in 10X Genomics read clouds (from molecules with an estimated N50 of 25 kb) were generated. Primary assembly contigs were scaffolded with chromosome conformation Hi-C data. Manual assembly curation corrected 974 missing/misjoins and removed 22 haplotypic duplications, reducing the assembly length by 2.1% and the scaffold number by 42.4%, and increasing the scaffold N50 by 198.1%.

**Figure 2.**
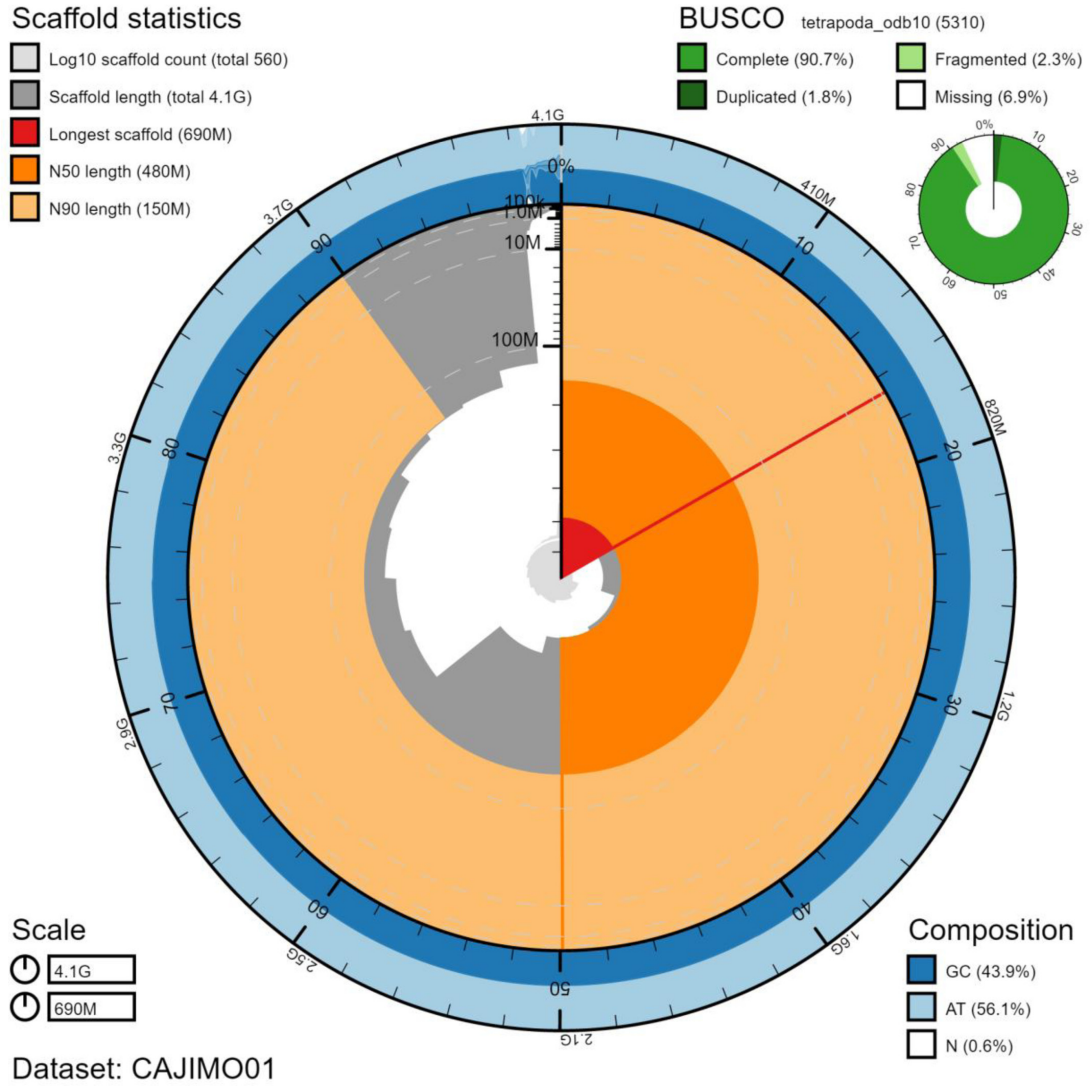
Genome assembly of *Rana temporaria*, aRanTem1.1: metrics. The BlobToolKit Snailplot shows N50 metrics and BUSCO gene completeness. The main plot is divided into 1,000 size-ordered bins around the circumference with each bin representing 0.1% of the 4,111,445,260 bp assembly. The distribution of scaffold lengths is shown in dark grey with the plot radius scaled to the longest scaffold present in the assembly (690,654,357 bp, shown in red). Orange and pale-orange arcs show the N50 and N90 scaffold lengths (481,763,206 and 153,779,893 bp), respectively. The pale grey spiral shows the cumulative scaffold count on a log scale with white scale lines showing successive orders of magnitude. The blue and pale-blue area around the outside of the plot shows the distribution of GC, AT and N percentages in the same bins as the inner plot. A summary of complete, fragmented, duplicated and missing BUSCO genes in the tetrapoda_odb10 set is shown in the top right. An interactive version of this figure is available at https://blobtoolkit.genomehubs.org/view/aRanTem1.1/dataset/CAJIMO01/snail.

The final assembly has a total length of 4.11 Gb in 555 sequence scaffolds with a scaffold N50 of 482 Mb ( Table 1). The majority, 99.5%, of the assembly sequence was assigned to 13 chromosomal-level scaffolds (numbered by sequence length) ( Figure 2– Figure 5; Table 2). The assembly has a BUSCO ( Simão *et al.*, 2015) v5.1.2 completeness of 90.7% using the tetrapoda_odb10 reference set. However, a BUSCO (v4.0.2) score of 95.2% using the same reference set was obtained for the annotated gene set of the aRanTem1.1 assembly (see section *Genome annotation*), indicating that the assembly has a high level of completeness and that some genes were missed during BUSCO analysis of the whole genome assembly. The values obtained for this assembly are higher than for a previous transcriptome assembly ( Ma *et al.*, 2018). While not fully phased, the assembly deposited is of one haplotype. Contigs corresponding to the second haplotype have also been deposited.

**Table 1.** Genome data for *Rana temporaria*, aRanTem1.1.

*Project accession data*	
Assembly identifier	aRanTem1.1
Species	*Rana temporaria*
Specimen	aRanTem1; NHMUK 2013.483
NCBI taxonomy ID	NCBI:txid8407
BioProject	PRJEB42239
BioSample ID	SAMEA7521635
Isolate information	Female, heart (genome assembly); kidney (Hi-C)
*Raw data accessions*	
PacificBiosciences SEQUEL I	ERR7012640-ERR7012642
10X Genomics Illumina	ERR6002771-ERR6002779, ERR6003050- ERR6003052
Hi-C Illumina	ERR6002780-ERR6002782
BioNano	ERZ3003200
*Genome assembly*	
Assembly accession	GCA_905171775.1
*Accession of alternate haplotype*	GCA_905171725.1
Span (Mb)	4,111
Number of contigs	2,411
Contig N50 length (Mb)	6.26
Number of scaffolds	554
Scaffold N50 length (Mb)	482
Longest scaffold (Mb)	691
BUSCO * genome score	C:90.7%[S:88.9%,D:1.8%],F:2.3%,M:6.9%,n:5310
*Genome annotation*	
Number of genes	36,124
Number of protein-coding genes	23,707
Average length of gene (bp)	52,818
Average number of exons per gene	14
Average exon size (bp)	273
Average intron size (bp)	9,757
BUSCO annotation score **	C:95.2%[S:92.8%,D:2.4%],F:0.6%,M:4.1%,n:5310

C= complete [S= single copy, D=duplicated], F=fragmented, M=missing, n=number of orthologues in comparison. *BUSCO scores based on the terapoda_odb10 BUSCO set using v5.1.2, run on the aRanTem1.1 genome assembly using BlobToolKit. A full set of BUSCO scores is available at https://blobtoolkit.genomehubs.org/view/aRanTem1.1/dataset/CAJIMO01/busco. **BUSCO scores based on the terapoda_odb10 BUSCO set using v4.0.2, run on the NCBI RefSeq annotation of the aRanTem1.1 genome assembly ( NCBI *Rana temporaria* Annotation Release 100).

**Figure 3.**
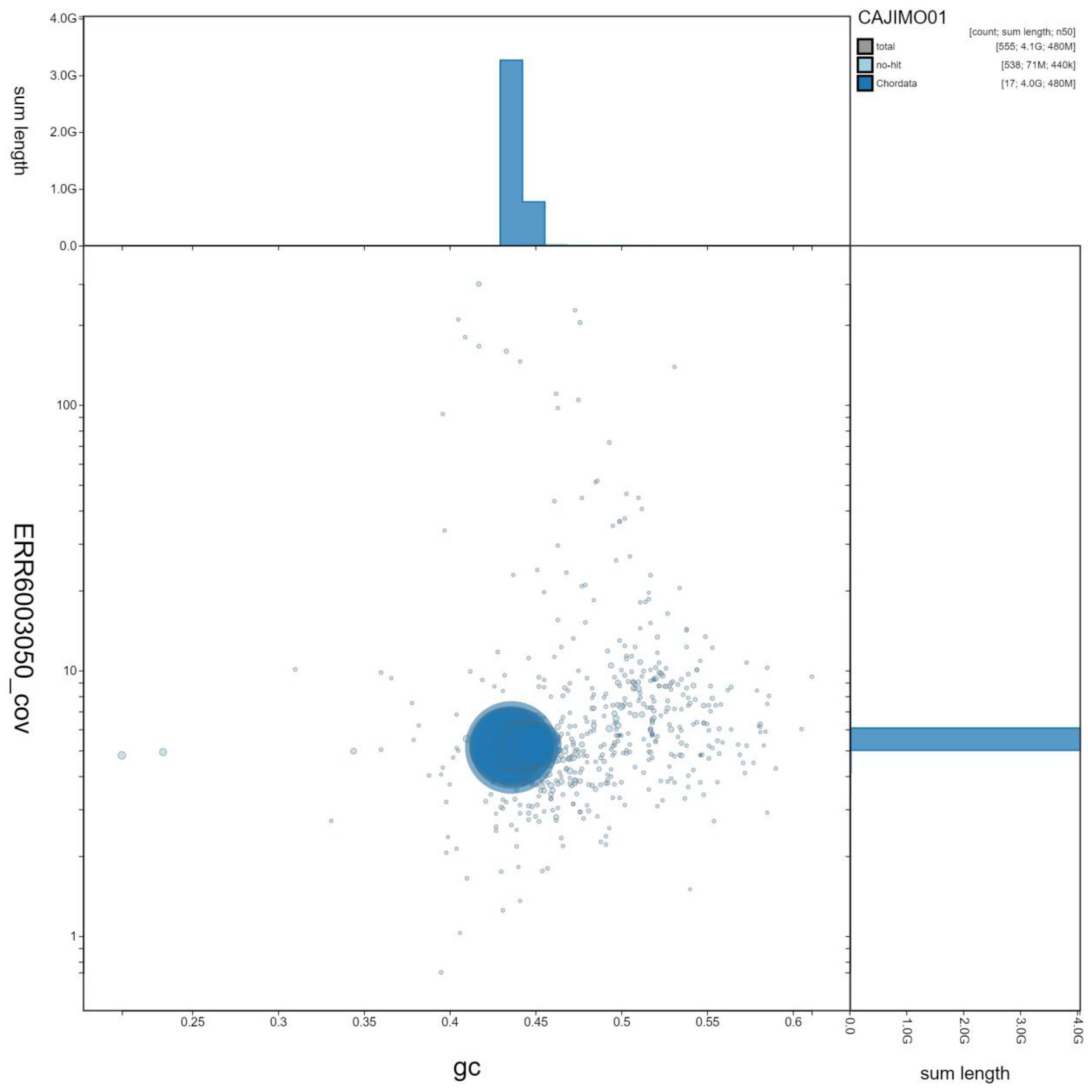
Genome assembly of *Rana temporaria*, aRanTem1.1: GC-coverage. BlobToolKit GC-coverage plot. Scaffolds are coloured by phylum. Circles are sized in proportion to scaffold length. Histograms show the distribution of scaffold length sum along each axis. An interactive version of this figure is available at https://blobtoolkit.genomehubs.org/view/aRanTem1.1/dataset/CAJIMO01/blob.

**Figure 4.**
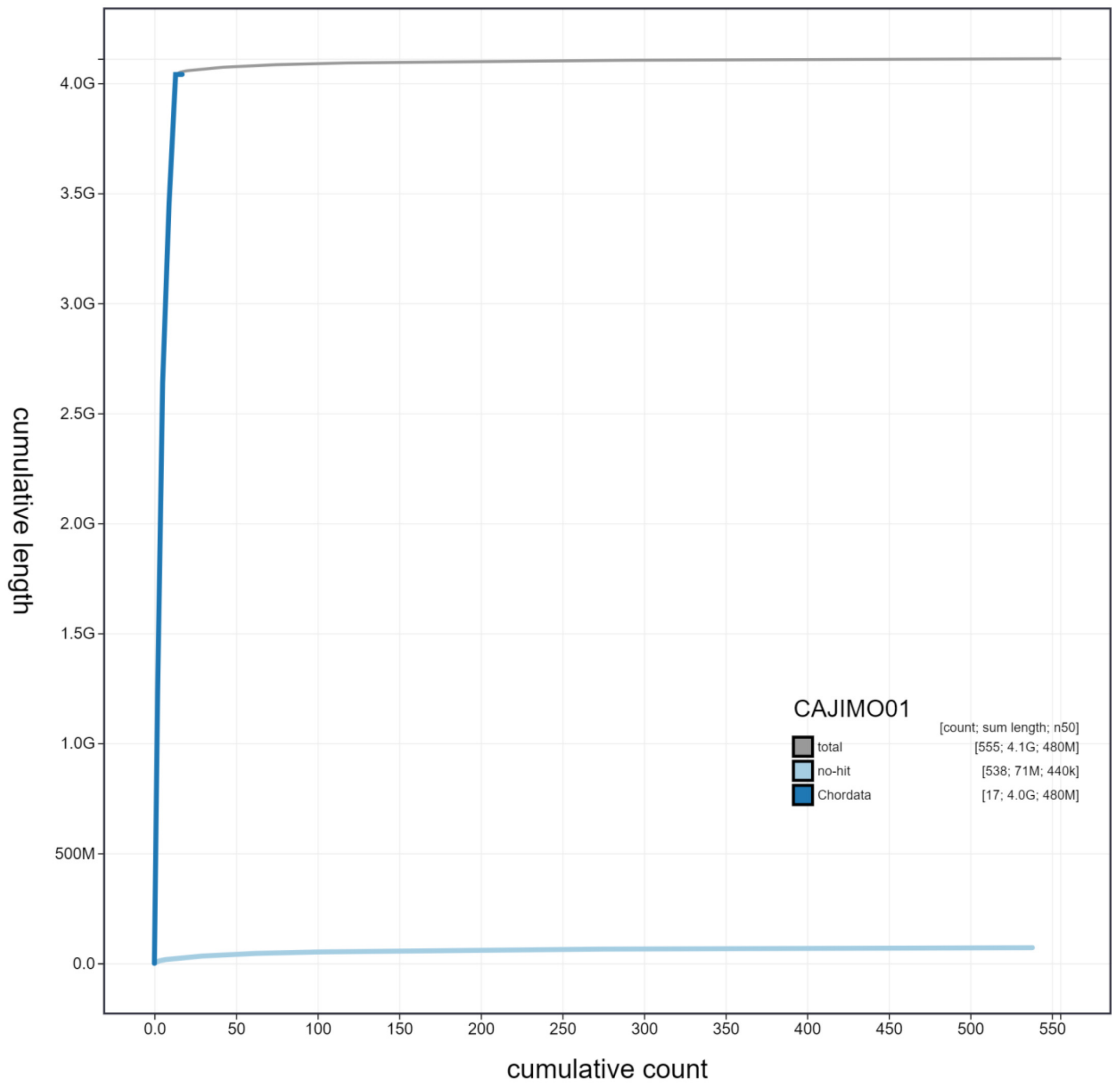
Genome assembly of *Rana temporaria*, aRanTem1.1: cumulative sequence. BlobToolKit cumulative sequence plot. The grey line shows cumulative length for all scaffolds. Coloured lines show cumulative lengths of scaffolds assigned to each phylum using the buscogenes taxrule. An interactive version of this figure is available at https://blobtoolkit.genomehubs.org/view/aRanTem1.1/dataset/CAJIMO01/cumulative.

**Figure 5.**
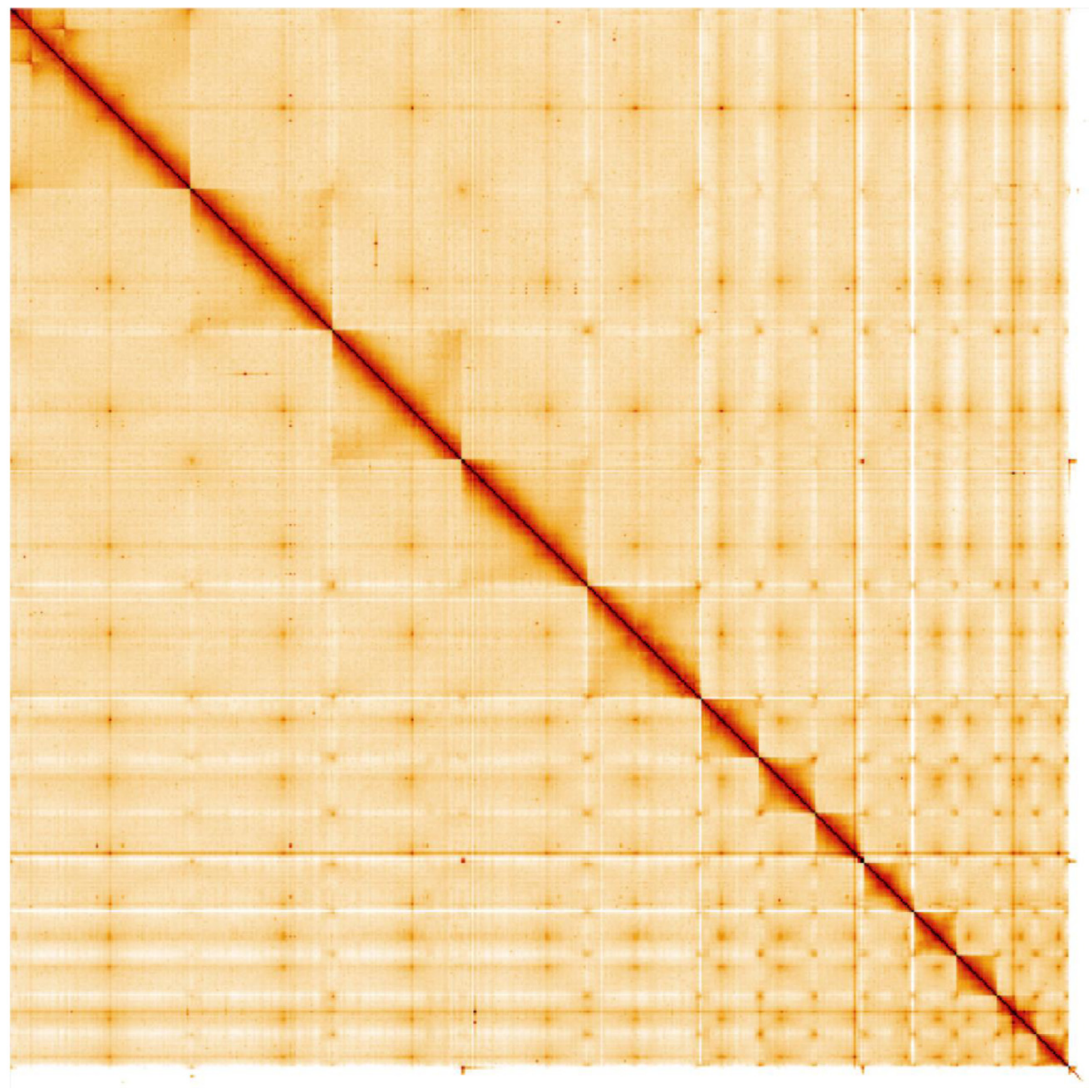
Genome assembly of *Rana temporaria*, aRanTem1.1: Hi-C contact map. Hi-C contact map of the aRanTem1.1 assembly, visualised in HiGlass. Chromosomes are arranged in size order from left to right and top to bottom.

**Table 2.** Chromosomal pseudomolecules in the genome assembly of *Rana temporaria*, aRanTem1.1.

INSDC accession	Chromosome	Size (Mb)	GC%
LR991680.1	1	690.65	43.8
LR991681.1	2	541.44	43.7
LR991682.1	3	495.42	44
LR991683.1	4	481.76	43.8
LR991684.1	5	429.35	43.9
LR991685.1	6	224.82	44.4
LR991686.1	7	212.59	44.7
LR991687.1	8	190.44	44.4
LR991688.1	9	184.30	44.4
LR991689.1	10	153.78	44.7
LR991690.1	11	164.33	44.8
LR991691.1	12	148.93	45.4
LR991692.1	13	121.98	45
LR991693.1	MT	0.02	40.5
-	Unplaced	71.62	47.9

## Genome annotation

The *R. temporaria* assembly was annotated by the NCBI Eukaryotic Genome Annotation Pipeline, an automated pipeline that annotates genes, transcripts and proteins on draft and finished genome assemblies. The annotation ( NCBI *Rana temporaria* Annotation Release 100; Table 1) was generated from transcripts and proteins retrieved from NCBI Entrez by alignment to the genome assembly, as described here ( Pruitt *et al.*, 2014).

## Methods

### Sample acquisition

A single female *R. temporaria* was collected from a stable, isolated population in the NHM Wildlife Garden, London, UK (latitude 51.49586, longitude -0.178622, elevation 17 m) by Jeffrey W. Streicher on 1 July 2015 ( Figure 1D). The specimen of *R. temporaria* (NHMUK 2013.483, Field ID: JWS 757) was 49.2 mm snout–vent length (determined using a Miyamoto digital calliper to the nearest 0.1 mm). The specimen was collected with permission from the NHM Wildlife Garden management team and is part of a long-term monitoring project run by the Department of Life Sciences and the Angela Marmont Centre for UK Biodiversity. It was humanely euthanised using a saturated solution of tricaine mesylate (MS-222). Multiple tissues including heart, thigh muscle, liver, eyes, kidney, ovaries, and intestines were sampled and placed in an ammonium sulfate-based RNA + DNA preservation buffer. After ~24 hours of storage at 4°C, the tissues were transferred to -80°C until they were sent for genome sequencing. Sample tissue has been accessioned by the Natural History Museum Molecular Collections Facility (NHMUK 2013.483).

### DNA extraction and sequencing

DNA was extracted from heart tissue in the Scientific Operations core of the Wellcome Sanger Institute using the Bionano Prep Animal Tissue DNA Isolation kit according to the manufacturer's instructions. Pacific Biosciences CLR long read and 10X Genomics read cloud sequencing libraries were constructed according to the manufacturers’ instructions. Hi-C data were generated from kidney tissue taken from the same animal using the Arima v2 Hi-C kit. Extraction and sequencing was performed by the Scientific Operations DNA Pipelines at the Wellcome Sanger Institute on Pacific Biosciences SEQUEL II (long-read) and Illumina HiSeq X (10X, Hi-C) instruments. DNA was labeled for Bionano Genomics optical mapping following the Bionano Prep Direct Label and Stain (DLS) Protocol and run on one Saphyr instrument chip flowcell.

### Genome assembly

Assembly was carried out following the Vertebrate Genome Project pipeline v1.6 ( Rhie *et al.*, 2021) with Falcon-unzip ( Chin *et al.*, 2016), haplotypic duplication was identified and removed with purge_dups ( Guan *et al.*, 2020) and a first round of scaffolding carried out with 10X Genomics read clouds using scaff10x. Hybrid scaffolding was performed using the BioNano DLE-1 data and BioNano Solve. Scaffolding with Hi-C data ( Rao *et al.*, 2014) was carried out with SALSA2 ( Ghurye *et al.*, 2019). The Hi-C scaffolded assembly was polished with arrow using the PacBio data, then polished with the 10X Genomics Illumina data by aligning to the assembly with longranger align, calling variants with freebayes ( Garrison & Marth, 2012) and applying homozygous non-reference edits using bcftools consensus. Two rounds of the Illumina polishing were applied. The mitochondrial genome was assembled using the mitoVGP pipeline ( Formenti *et al.*, 2021). The assembly was checked for contamination and corrected using the gEVAL system ( Chow *et al.*, 2016; Howe *et al.*, 2021). Manual curation was performed using evidence from Bionano (using the Bionano Access viewer), using HiGlass ( Kerpedjiev *et al.*, 2018) and Pretext, as described previously ( Howe *et al.*, 2021). Figure 2– Figure 4 and BUSCO values were generated using BlobToolKit ( Challis *et al.*, 2020). Table 3 includes a list of software tools used.

**Table 3.** Software tools used.

Software tool	Version	Source
Falcon-unzip	falcon-kit 1.4.2	Chin *et al.*, 2016
purge_dups	1.0.0	Guan *et al.*, 2020
SALSA2	2.2-14-g974589f	Ghurye *et al.*, 2019
scaff10x	4.2	https://github.com/wtsi-hpag/Scaff10X
Bionano Solve	3.3_10252018	https://bionanogenomics.com/downloads/bionano-solve/
arrow	gcpp 1.9.0-SL-release-8.0.0+1-37-gd7b188d	https://github.com/PacificBiosciences/GenomicConsensus
longranger align	2.2.2	https://support.10xgenomics.com/genome-exome/software/ pipelines/latest/advanced/other-pipelines
freebayes	1.3.1-17-gaa2ace8	Garrison & Marth, 2012
bcftools consensus	1.9-78-gb7e4ba9	http://samtools.github.io/bcftools/bcftools.html
mitoVGP		Formenti *et al.*, 2021
HiGlass	1.11.6	Kerpedjiev *et al.*, 2018
PretextView	0.1	https://github.com/wtsi-hpag/PretextView
gEVAL	N/A	Chow *et al.*, 2016
BlobToolKit	2.6.1	Challis *et al.*, 2020

### Ethical/compliance issues

The materials that have contributed to this genome note were supplied by a Tree of Life collaborator. The Wellcome Sanger Institute employs a process whereby due diligence is carried out proportionate to the nature of the materials themselves, and the circumstances under which they have been/are to be collected and provided for use. The purpose of this is to address and mitigate any potential legal and/or ethical implications of receipt and use of the materials as part of the research project, and to ensure that in doing so we align with best practice wherever possible.

The overarching areas of consideration are:

- Ethical review of provenance and sourcing of the material;
- Legality of collection, transfer and use (national and international).

Each transfer of samples is undertaken according to a Research Collaboration Agreement or Material Transfer Agreement entered into by the Tree of Life collaborator, Genome Research Limited (operating as the Wellcome Sanger Institute) and in some circumstances other Tree of Life collaborators.

## Data availability

European Nucleotide Archive: Rana temporaria (common frog). Accession number PRJEB42239: https://identifiers.org/ena.embl:PRJEB42239

The genome sequence is released openly for reuse. The *R. temporaria* genome sequencing initiative is part of the Darwin Tree of Life (DToL) project and the Vertebrate Genomes Project. All raw sequence data and the assembly have been deposited in INSDC databases. Raw data and assembly accession identifiers are reported in Table 1.

## Author information

Members of the Wellcome Sanger Institute Tree of Life programme collective are listed here: https://doi.org/10.5281/zenodo.5377053.

Members of Wellcome Sanger Institute Scientific Operations: DNA Pipelines collective are listed here: https://doi.org/10.5281/zenodo.4790456.

Members of the Tree of Life Core Informatics collective are listed here: https://doi.org10.5281/zenodo.5013542.

Members of the Darwin Tree of Life Consortium are listed here: https://doi.org/10.5281/zenodo.4783559.
