# Supplementary material for: The genome sequence of the common frog, Rana temporaria Linnaeus 1758
Source: Wellcome Open Res. 2021 Oct 22;6:286. [Version 1] doi: 10.12688/wellcomeopenres.17296.1 (PMC8790709; doi:10.12688/wellcomeopenres.17296.1)
Supplement: Peer review history

# Peer review: Reviewer response for version 1

**DOI:** 10.21956/wellcomeopenres.19123.r48171

**Published:** 2022-01-25

**Authors:** Roberto Márquez

**Affiliations:** 1 Department of Ecology and Evolutionary Biology, University of Michigan, Ann Arbor, MI, USA

This report presents an annotated, chromosome-level genome assembly for the European common frog, *Rana temporaria*. Using a combination of sequencing strategies and technologies the authors produced a remarkably contiguous and complete assembly for an amphibian, which will certainly facilitate research on this species, and amphibians in general. The report is complete and thorough, and should be able to be indexed after a couple of very minor adjustments, detailed below:

1. According to Table 1 Pac Bio Sequel I was used, but in the Methods section Sequel II is mentioned. Please confirm which of the two technologies was used. 
2. The use of BioNano technology is not mentioned in the Genome sequence report section. I suggest including it for completeness. 
3. Although the methodological details provided are very thorough and would mostly allow for replication of the study, I encourage the authors to provide their code in a public repository. This would make the study 100% replicable, and may help other researchers wrestling with amphibian genomes advance their own assembly projects. 

Are sufficient details of methods and materials provided to allow replication by others?

Partly

Is the rationale for creating the dataset(s) clearly described?

Yes

Are the datasets clearly presented in a useable and accessible format?

Yes

Are the protocols appropriate and is the work technically sound?

Yes

Reviewer Expertise:

Evolutionary genetics and systematics of neotropical amphibians.

I confirm that I have read this submission and believe that I have an appropriate level of expertise to confirm that it is of an acceptable scientific standard.

# Peer review: Reviewer response for version 1

**DOI:** 10.21956/wellcomeopenres.19123.r46611

**Published:** 2021-11-10

**Authors:** Peng Xu, Baohua Chen

**Affiliations:** 1 College of Ocean and Earth Sciences, Xiamen University, Xiamen, China

Streicher *et al. *provided the first complete reference genome and corresponding annotation of common frog with extremely high quality. The workflow used in generating this dataset was very appropriate and normative. This work provides a very valuable genetic resource for further relative studies. I appreciate their efforts.

 The only flaw of this data note, in my opinion, was that a few importance details in method and report had been missing:

- First, extra information of genome annotation results should be provided besides a single table (Table 1) including, but not limited to, numbers and lengths of genes, ncRNAs and exons. Moreover, due to the extremely large size of this species, the number and classification of repeat sequences were very informative for other researchers. The author should also provide this.
- Second, I am very curious about how the authors combined the Hi-C data and Bionano data. Although a reference had been cited which can illuminate this, I think the authors still should provide a brief introduction in his report for convenience. In addition, the “Genome annotation” section seems like should be placed in “Methods” section.

Are sufficient details of methods and materials provided to allow replication by others?

Partly

Is the rationale for creating the dataset(s) clearly described?

Yes

Are the datasets clearly presented in a useable and accessible format?

Yes

Are the protocols appropriate and is the work technically sound?

Yes

Reviewer Expertise:

Fish genetics and genomics

We confirm that we have read this submission and believe that we have an appropriate level of expertise to confirm that it is of an acceptable scientific standard.
